# [Corrigendum] miR‑27a promotes proliferation, migration, and invasion of colorectal cancer by targeting FAM172A and acts as a diagnostic and prognostic biomarker

**DOI:** 10.3892/or.2024.8742

**Published:** 2024-04-24

**Authors:** Wenjun Liu, Kai Qian, Xing Wei, Haijun Deng, Bei Zhao, Qing Chen, Jinqian Zhang, Hao Liu

Oncol Rep 37: 3554–3564, 2017; DOI: 10.3892/or.2017.5592

Following the publication of this article, an interested reader drew to the authors' attention that the western blots in Fig. 4B on p. 3560 and Fig. 6B on p. 3562 shared remarkably similar data (including both the GAPDH and the FAM172A blots in Fig. 4B), such that these data were likely to have been derived from the same original source. Upon asking the authors to provide an explanation, the authors realized that these errors inadvertently arose during the process of assembling these figures. Due to a mislabelling of the files, representative blots for FAM172A and GAPDH were chosen incorrectly for Fig. 4B. The authors had retained their original data, however, and were also able to present to the Editorial Office for our perusal the uncropped versions of their western blots, which resolved any other potential issues of anomalies associated with the data.

The revised version of Fig. 4, now showing alternative data for Fig. 4B, is shown on the next page (note that, in the repeated experiment, relative to the original version of this figure the miR-27a, miR27a-inhibitor and negative control experiments were run on different lanes of the gel). Also note that the errors made in terms of assembling the data in Fig. 4 did not greatly affect either the results or the conclusions reported in this paper, and all the authors agree to the publication of this corrigendum. The authors regret that these errors went unnoticed prior to the publication of their article, are grateful to the Editor of *Oncology Reports* for granting them this opportunity to publish a corrigendum, and apologize to the readership for any inconvenience caused.

## Figures and Tables

**Figure 4.**
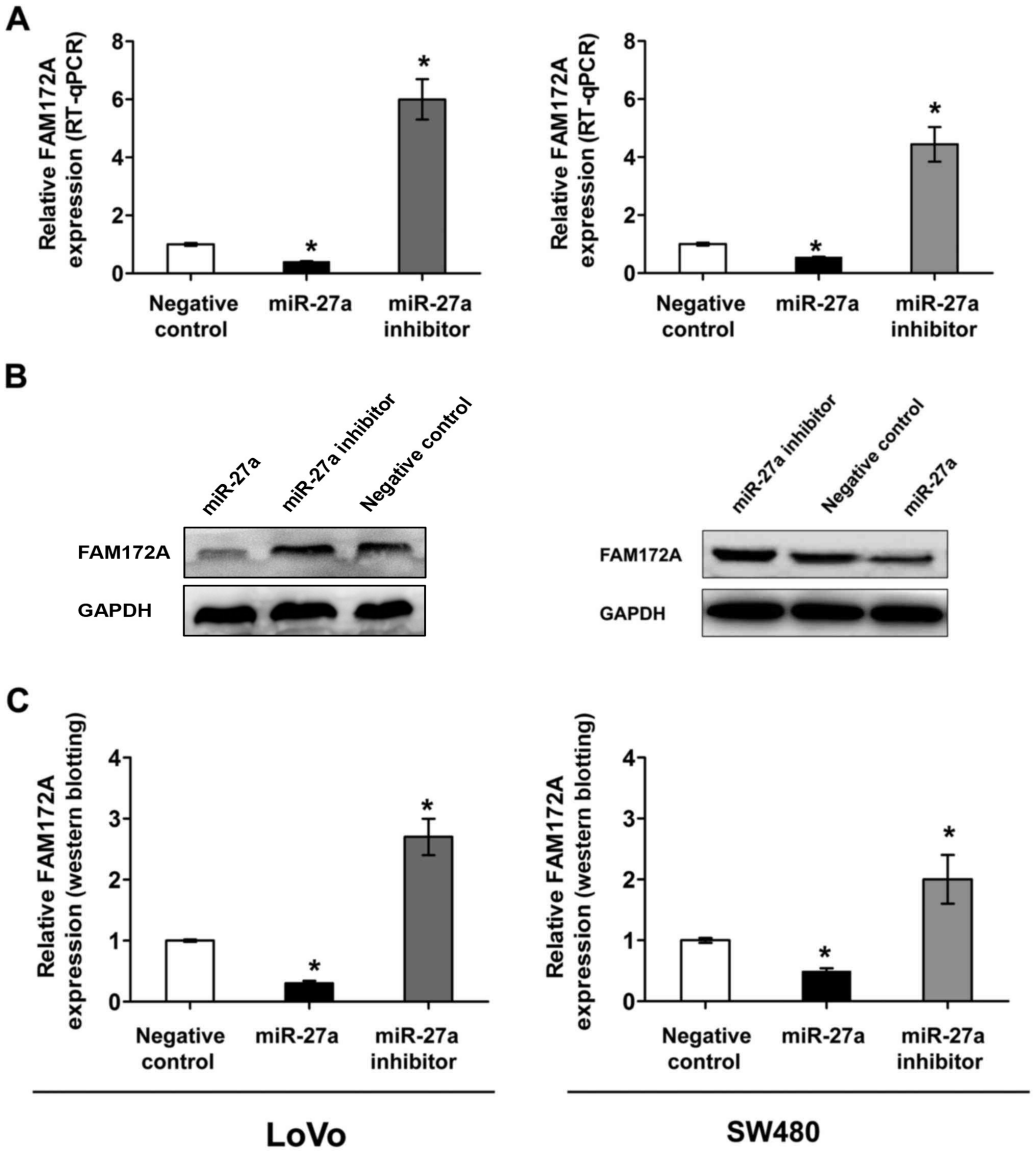
miR-27a negativly regulated the FAM172A expression in CRC cell lines. (A,B) The mRNA levels of FAM172A expression was examined by RT-qPCR in the LoVo and SW480 colorectal cell lines following transfection with miR-27a mimic, miR-27a inhibitor and cont-miR, *P<0.05. (C) The protein levels of FAM172A expression was examined by western blotting in the LoVo and SW480 colorectal cell lines following transfection with miR-27a mimic, miR-27a inhibitor and cont-miR, *P<0.05.

